# Digital Transformation in Thoracic Surgery: a survey among the European Society of Thoracic Surgeons

**DOI:** 10.1093/icvts/ivae119

**Published:** 2024-06-28

**Authors:** Nora Mayer, George Sotiropoulos, Nuria Novoa, Niccolo Daddi, Hasan Batirel, Nizar Asadi

**Affiliations:** Department of Thoracic Surgery, Harefield Hospital, Guy’s and St. Thomas’ NHS Foundation Trust, London, UK; Department of Thoracic Surgery, Athens Naval Hospital, Athens, Greece; Thoracic Surgery Department, University Hospital Puerta de Hierro-Majadahonda, Majadahonda, University of Salamanca. Biomedical Institute of Salamanca (IBSAL), Salamanca, Spain; Department of Thoracic Surgery, IRCCS Azienda Ospedaliera Universitaria di Bologna, Bologna, Italy; Department of Thoracic Surgery, Marmara University School of Medicine, Istanbul, Turkey; Department of Thoracic Surgery, Harefield Hospital, Guy’s and St. Thomas’ NHS Foundation Trust, London, UK

**Keywords:** Digital transformation, Thoracic surgery, Surgical training, Telemedicine, e-Health-system, Patient care

## Abstract

**OBJECTIVES:**

Digital transformation has drastically changed the surgical sector, but few is known about its impact on thoracic surgical practice. The aim of this paper is to report the European Society of Thoracic Surgeons (ESTS) survey results, assessing the impact of and the need for Digital Transformation in Thoracic Surgery.

**METHODS:**

A 23-item survey was designed by the ESTS Digital Transformation Working Group to assess the impact of and the need for Digital Transformation in Thoracic Surgery. All ESTS members (1668) were invited to complete the survey between 13 March and 21 May 2022 anonymously. Data analysis was descriptive calculating frequencies and percentages. Group comparison was done using chi-square test.

**RESULTS:**

The response rate was 6.3%. Surgeons from 26 European countries participated of which more than 80% were based in academic hospitals. The impact of digital transformation was rated very important (43.8%) and fundamental (22.7%) in more than two-thirds of the cases, regardless of surgeons’ age. None of the participants felt that digital transformation was of no importance and more than 85% had implemented digital platforms in their direct patient care. Almost 90% of the surgeons, currently not using digital platforms for training and education, would consider introducing them. About 70% were at least ‘somewhat satisfied’ with their current engagement in Digital Transformation in Thoracic Surgery.

**CONCLUSIONS:**

Digital transformation seems to play a major role across European Thoracic Surgery departments in direct patient care, professional networking and surgical training. However, overall satisfaction with the current status of Digital Transformation in Thoracic Surgery was rather reserved, implying the need to increase the implementation of digital solutions in the latter.

## Graphical abstract

**Figure:**
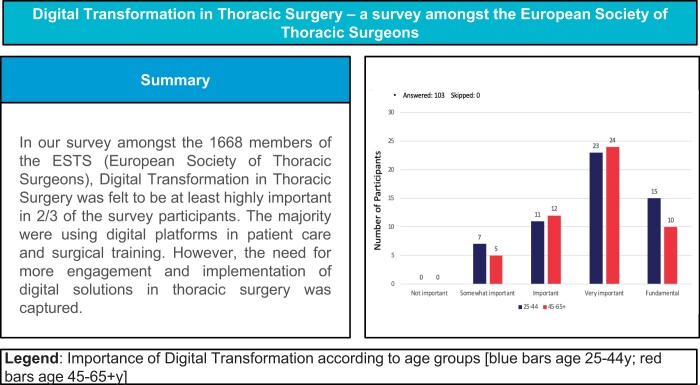

## INTRODUCTION

Digitalization has been rapidly transforming health care over the last few decades. The integration of digital devices and solutions such as remote patient monitoring, exercise trackers, e-learning platforms, computer-aided visualization and artificial intelligence in everyday surgeons’ life and patient care, especially during the challenging Corona virus disease 2019 (COVID-19) pandemic has had a huge impact on the way surgery is practiced and will continue to play a huge role on how we deliver healthcare in the future [1, 2].

Digital transformation extends beyond the digitalization of several aspects of modern medicine. It represents a complex process of a cultural change to integrate technologies, create new operational models and reorganize medical services to achieve the optimal health care provision [3].

The European Society of Thoracic Surgery (ESTS) represents a mixture of practicing thoracic surgeons at various stages of education in different European and several non-European countries. Each country with different healthcare systems and variable levels of digitalization integrated into patient care and surgical education.

However, no specific data on the degree of digital penetration among thoracic surgeons have been published. Neither information about the use of digital training platform or tele-mentoring in thoracic surgery has been provided.

On behalf of the ESTS, the Digital Transformation Working Group was founded. The group developed the current survey. It was designed to understand the current status of Digital Transformation in General Thoracic Surgery across the ESTS. The aim of this paper is to report the results of the survey sent to the ESTS membership in the spring of 2022.

## MATERIALS AND METHODS

### Ethical statement

All ESTS members (*N* = 1668) included in the mailing list of the society who had consented to be contacted, received an email inviting them to complete an electronic survey. The survey was open from 13 March through 21 May 2022. Anonymous responses were collected through a link to a commercially available platform (www.surveymonkey.com). The survey was designed by the ESTS Digital Transformation Group and approved by the ESTS councils. The survey was sent out once within the above-mentioned period. The data underlying this article will be shared on reasonable request to the corresponding author.

### Survey design

A 23-item survey investigating the role digital transformation is currently playing in Thoracic Surgery departments across Europe was designed and distributed with an introductory letter explaining the purpose of the survey (Supplementary Material). There were no exclusion criteria. All responses were voluntary and anonymous. The ESTS represents more than 1600 members coming from 91 different countries all over the world.

The questionnaire was developed by members of the ESTS Digital Transformation Group and subsequently submitted for revision and approval to the ESTS Board of Directors. The questions were designed to retrieve objective data on the participants’ demographics, training and professional status, their experience with digital transformation projects and usage of digital solution applications in their department. In the 2nd part of the survey, the respondents were asked to rate the importance of Digital Transformation in Thoracic Surgery and state their satisfaction with the current availability and infrastructure of the same.

The items were structured in decision questions, standard five-point Likert items to strongly agree through strongly disagree with a given statement and feedback questions. The results of each question are presented in bar charts and tables. The complete survey and results are available in the Supplementary Material 1 (Original survey). In the last question, participants also had the chance to express their thoughts in an open question.

### Statistical analysis

Data was collected and analysed using IBM^®^ SPSS Statistics. Primary data analysis was descriptive calculating frequencies and percentages. Group comparison was performed using chi-square test. A *P*-value of <0.05 was considered statistically significant.

## RESULTS

Out of the 1668 ESTS members who were invited to participate, 105 responded (6.3% response rate). Email delivery rate reported by the survey platform was 99.2%. Item completion rate was 87%. The survey was sent to the participants on 13 March with more than 70% of the participants replying within the 1st week.

### Demographics

**Figure 1:**
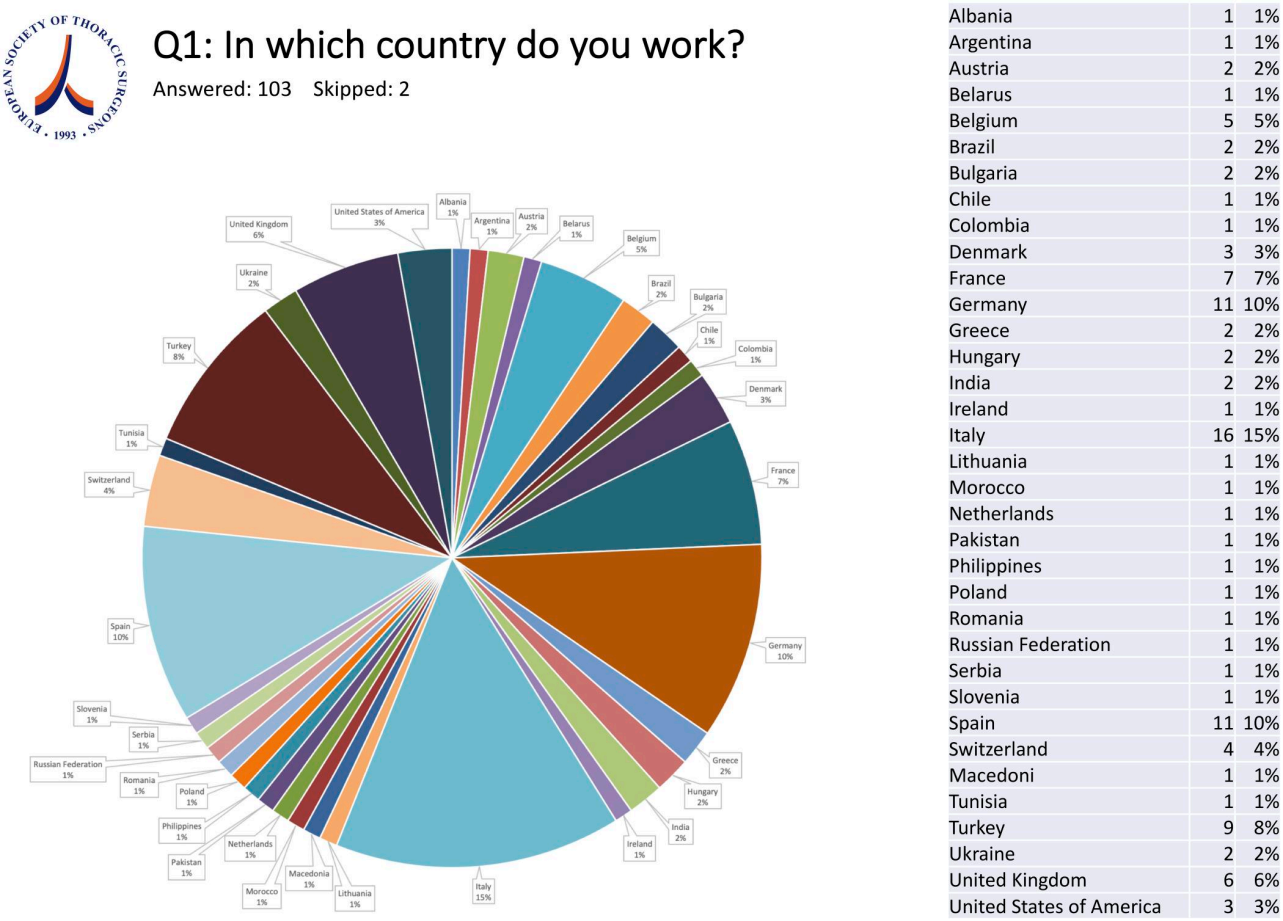
Distribution of countries of participating ESTS members.

Thoracic surgeons working in 26 countries responded to the survey of which 20 were European countries, 3 Asian countries, 1 from North America and 2 from South America (Fig. 1).

Responders were quite evenly distributed between different age groups. Twenty-six (24.76%) of them were between 25 and 34 years old, 29 (27.62%) were between 35 and 44, 30 (28.57%) were between 45 and 54 and 20 (19%) were above 55 years old; 76.2% (80) were male. The largest group of the participants stated to be in their 1st decade of individual practise (34.5%). Eighteen (17.14%) were still in training, whereas 27 (25.71%) were in their 2nd and 17 (16.19%) were in their 3rd decade as consultant/staff surgeons. Only 7 (6.67%) had been practicing individually for more than 30 years. Eighty-four (80.8%) were working in academic hospitals, whereas 10 (9.6%) were working in supra-regional/non-academic hospitals and 9 (8.6%) in regional/non-academic hospitals.

### Digital Transformation in Thoracic Surgery

**Figure 2:**
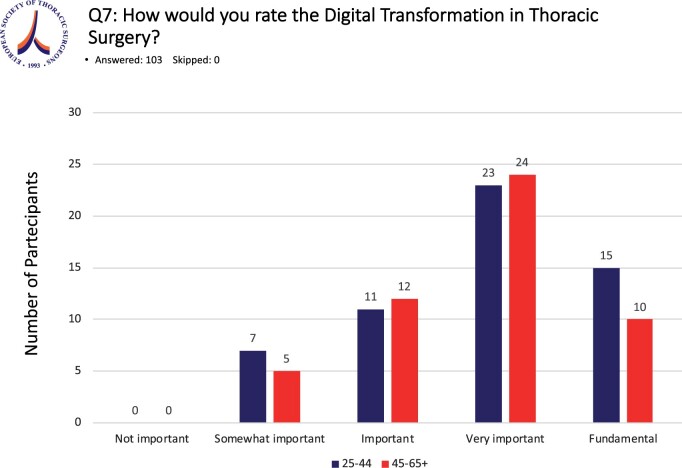
Importance of Digital Transformation in Thoracic Surgery. Respondents were divided into 2 groups according to age, group A (blue, 25–44 years old), group B (red, 45–65+ years old). There is no statistical difference between the perception of the importance of Digital Transformation in Thoracic Surgery among the participating surgeons.

When asked about the importance of Digital Transformation in Thoracic Surgery, none of the respondents felt it was ‘not important’ and 12 (11.4%) replied it was ‘somewhat important’. Twenty-three (21.9%) considered digital transformation as being ‘Important’, 46 (43.8%) ‘very important’ and 24 (22.9%) ‘fundamental’. To assess potential differences in the perception of the importance of Digital Transformation in Thoracic Surgery between different age groups, the respondents were divided into 2 groups for analysis. Group A consisted of those being between 25 and 44 years old (*n* = 56, 52%) and Group B between 45 and 65+ (*n* = 51, 48%). In thoracic surgery, 26.8% of the surgeons in Group A answered that digital transformation was of ‘fundamental’ importance in Thoracic Surgery compared to 19.6% in Group B. Group A respondents considered digital transformation as ‘very important’ (41.1%), ‘important’ (19.6%) and ‘somewhat important’ (12.5%). In Group B, the corresponding response percentages were 47.1%, 23.5% and 9.8% (Fig. 2). There was no statistical difference between the responses of the 2 groups (*P* = 0.76).

The overwhelming majority (85.7%) of the participants answered ‘yes’ to the question of whether they were using some kind of digital platform for direct patient care. From those who answered ‘yes’, 8 (8.8%) responded that the best value of their digital platform lies in patient pre-assessment, 17 (18.7%) in patient care during hospital stay, 9 (8.8%) in post-operative care and 57 (62.6%) in all 3 areas. Only one (1%) surgeon responded that its best value did not lie in any of these 3 areas.

To extract data for clinical studies, 60 participants (57.7%) used a local database, such as Excel or Google Drive, 68 (65.4%) a hospital-based database, 28 (26.9%) a national database and 19 (18.27%) the ESTS database.

### Training and education

**Figure 3:**
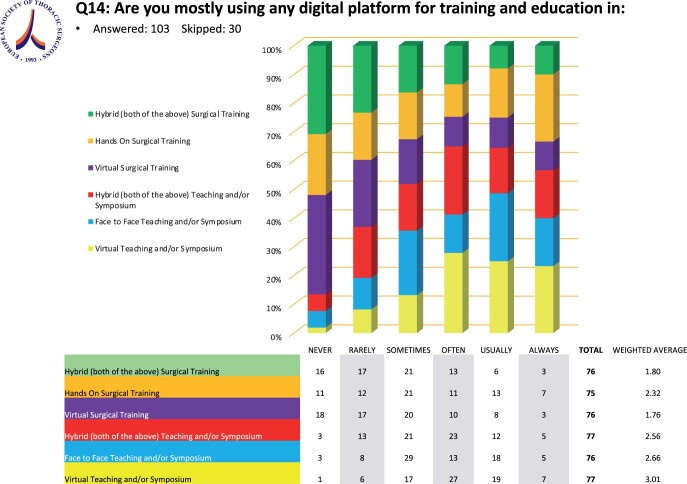
Distribution of the use of digital platforms in training and education.

Sixty-one (58.6%) surgeons reported using a digital platform for training and education, while 48 (38.5%) were not and 3 (2.9%) did not know whether a digital platform was in use in their department. Among the group that was not using any digital platform in training, 43 (89.6%) claimed that they would consider introducing a platform solution in their department. The group currently using a digital platform for training and education mostly uses it for ‘virtual teaching and/or symposium’ and ‘face-to-face teaching and/or symposium’ (Fig. 3).

### Networking and communication

**Figure 4:**
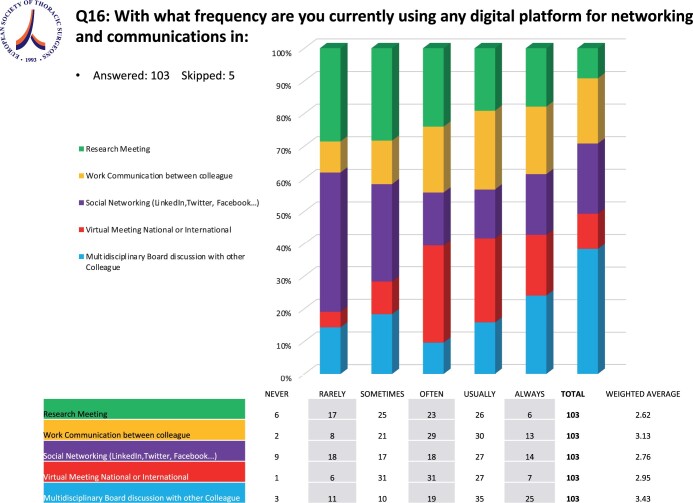
Digital platforms in networking and communication in thoracic surgery.

When asked whether they were using any digital platform for networking and communications, the vast majority of the participants, 96 (91.4%), answered ‘Yes’, with only 9 (8.6%) answering ‘No’. The respondents answering ‘Yes’ would use digital platforms more frequently for ‘Multidisciplinary Board discussion with other Colleagues’ and ‘Work Communication between Colleagues’ (Fig. 4).

The most popular app for social networking among the respondents was LinkedIn with 63 (64.3%) users. Thirty-three (33.7%) used Facebook, 30 (30.6%) Instagram, 22 (22,4%) Twitter, 14 (14.3%) Telegram and 18 (18.3%) other applications. The next question was about the use of an institutional app for work communication between colleagues. Forty (38.1%) were using one and 35 (33.3%) did not use an institutional app. Those who were not using an institutional app were either using a commercially available app for General Data Protection Rating (GDPR)-conform communications or an institutional email service and 30 (28.6%) did not use any.

Finally, when asked whether participants would consider using a specified digital platform for healthcare professionals the overwhelming majority, 89 (85.6%), answered ‘Yes’, 11 (10.6%) ‘Didn’t Know’ and 4 (3.8%) stated ‘No’.

### Purpose of the ESTS Digital Transformation Working Group

**Figure 5:**
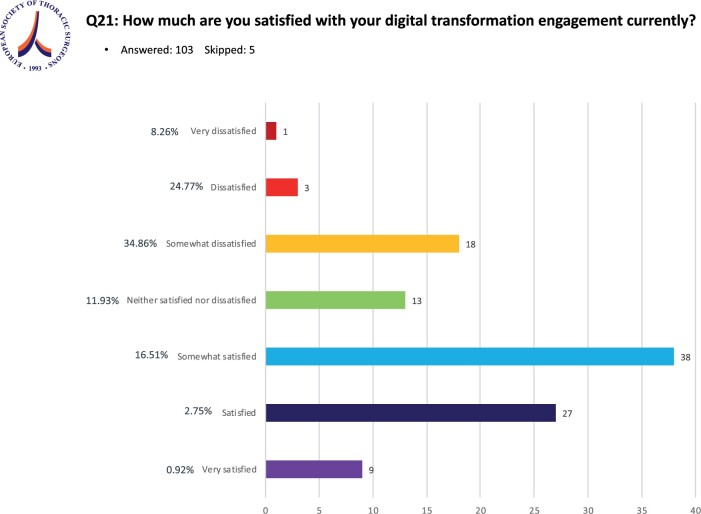
Status of current satisfaction with engagement in digital transformation.

In terms of the respondents’ satisfaction of their current digital transformation engagement 9 (8.6%) surgeons were ‘very satisfied’, 26 (24.8%) ‘satisfied’, 35 (33.3%) ‘somewhat satisfied’, 13 (12.4%) ‘Neither satisfied or dissatisfied’, 18 (17.1%) ‘somewhat satisfied’, 3 (2.9%) ‘dissatisfied’ and 1 (0.9%) ‘very dissatisfied’ (Fig. 5).

The question ‘What do you think this ESTS Digital Transformation Working Group should be focusing on?’ was answered with a clear statement of 58 (59.8%) feeling that the working group should focus on ‘Introduction of digital platforms for teaching and training’, followed by the ‘Introduction of digital platforms for patient healthcare’ by 23 (23.7%) of the respondents, 15 (15.5%) chose ‘Introduction of digital platforms for clinical communication and social media’ and 1 (1%) responded with ‘Other’, not further specified in this survey.

The last question was an open question to add any personal comments. We received a total of 9 comments. Four (44.4%) emphasized the need for digital transformation and development of tools for professional exchange between colleagues. One (1.1%) expressed the opinion that the ESTS should focus on uniform training across different countries and not digitalization, 2 (2.2%) emphasized personal or national involvement in digital transformation programs and 2 (2.2%) were supportive comments towards the ESTS Digital Transformation Group.

## DISCUSSION

In this ESTS survey, the impact of Digital Transformation on Thoracic Surgery was rated very important (43.8%) and fundamental (22.7%) in more than two-thirds of the participating surgeons from 26 European countries, regardless of their age group. Since more than 75% of the survey respondents were European, the survey results cannot be considered representative of a worldwide view on the aforementioned and have to be interpreted in a European context.

Most of the respondents (85%) had already implemented digital solutions in their direct patient care and almost 90% of the surgeons, who were not currently using digital platforms for training and education, would consider introducing them. About 70% of the surgeons were at least ‘somewhat satisfied’ with their current engagement in digital transformation.

Thoracic Surgery is a rapidly evolving specialty. The Digital Transformation of Thoracic Surgery, meaning ‘a transformation of daily practice that requires fundamental organizational change as well as the implementation of digital technologies’, has become a reality. Digital solutions like artificial intelligence programs, preoperative 3D-reconstruction planning, implementation of surveillance apps for perioperative patient care and remote surgical teaching are fully integrated into our schedules [4–6].

Not long ago, innovative digital solutions initially found their way into medicine, blazingly fast revolutionizing all different healthcare sectors as exemplarily classified by the World Health Organization [7, 8]. As experience has shown, there is a considerable market for digital solutions in surgery, especially day case surgery, and perioperative care [9]. Especially dealing with the recent COVID-19 pandemic from 2019 to 2022 with a disruption of the until then known thoracic surgical service, a temporary transformation of the health care system to a predominantly remote organization to guarantee patient care, has emphasized the importance of Digital Transformation in Thoracic Surgery [10, 11].

Digital solutions nowadays have been widely implemented in primary patient care where they have been shown to improve patient engagement and outcome via telehealth [2, 12–14]. Moreover, digital platforms increasingly play a major role in clinical academic research, surgical training and are regularly used for communication and networking between healthcare professionals [15, 16]. While on-site surgical training was temporarily critically disrupted by redeployment of the trainees and pandemic restrictions, virtual training was successfully implemented [17–19]. To guarantee continuity of health care services, especially during the COVID-19 pandemic, virtual clinics, multidisciplinary team meetings and augmented reality in live surgery in virtual cooperation with surgeons on a different continent became daily business [11, 20].

Our study evaluated the ESTS members’ perspective on the importance and current status of Digital Transformation in Thoracic Surgery [10]. We could show that more than half of the respondents, and interestingly independent of their age group, felt, that digital transformation was very important or even a fundamental part of Thoracic Surgery. In general, younger generations are more commonly described as early adopters with regard to technological innovations and might be suspected to be the group being more engaged in digital transformation. However, our study results revealed, that further advanced surgeons in Group B (Fig. 2), aged 45–65+, gave digital transformation the same priority and importance in their practice compared to the younger age group (Group A) of thoracic surgeons.

In daily routine in the respondents’ hospitals and practices, multidisciplinary team meetings were basically exclusively run via digital platforms as reported. Digital platforms as well seem to have been implemented in some thoracic surgical departments in virtual and hands-on surgical training as well as in symposia [21, 22]. However, on the other hand, close to 40% of the departments stated that digital platforms played no role in their training program. The majority, precisely more than 80% of the respondents who were not using a virtual surgical training platform, claimed they would use one. This answer legitimately raises questions. What prevents certain Thoracic Surgery departments from implementing digital platforms for teaching and training even though there is an unequivocal wish to do so? Our survey included responders from international healthcare systems, mainly European ones, many of which might be facing budget cuts in the healthcare sector. Moreover, as reported in the Annual European eHealth Survey 2019 [23], lack of funding is definitely a factor preventing the digitalization of medical training. Another reason against further digitalization certainly addresses concerns regarding the safety of patient data, as many institutions do not have access to applications designed for medical use complying with the GDPR of the respective countries regarding health-related data.

What gave us food for thought in addition was the discrepancy of many respondents agreeing that digital transformation was at least important, if not very important or fundamental in thoracic surgery, whereas only a third was convincingly satisfied with their current engagement in digital transformation (Fig. 5). Another 33.3% were ‘somewhat satisfied’, creating the impression of the need to improve the current engagement. Reasons for a not entirely satisfying up to dissatisfying engagement in digital transformation in certain thoracic surgery units were not further evaluated in this survey but might be related to slow uptake and adoption of digital transformation in health care due to institutional limitations [24, 25]. Further problems identified as barriers to more engagement include liability concerns, costs, usability and policy problems through to acceptability to professionals and patients [26].

Having seen the discrepancy between the perceived importance of digital transformation and the status of satisfaction, the launch of the ESTS Digital Transformation Working Group in 2022 to assess the current situation and support the implementation of digital solutions in thoracic surgery departments across the world was inevitable. Moreover, through this survey, we could identify the clear assignment for the society to pioneer the structured inclusion of digital transformation for teaching and training in the ESTS, as requested by nearly 60% of the survey participants. This leads to the question of how much digitalization is necessary and welcome as well as how to guarantee patient (data) safety without having much-published evidence for the use of digital solutions in thoracic surgery. In this regard, the ESTS will have to be wisely learning from early-adapting countries like the UK, where Public Health England lately even published their Digital-first public health strategy [27].

What certainly needs to be considered before drawing a conclusion is the fact, that this survey was a purely one-sided evaluation from a physician’s point of view on Digital Transformation in Thoracic Surgery. Without having clear evidence of the effect on thoracic patients’ satisfaction with being moved to perioperative remote care and thoracic training being shifted to being run via digital solutions, the ESTS Digital Transformation Working Group will have to further evaluate the impact of digital transformation on both patient care and surgical training [28].

### Limitations

This study has potential limitations. First, the multinationalism of the respondents working in various healthcare systems may imply differences in the level of digitalization and access to digital solutions which may bias the responses of the participants. No differentiation was made between any correlation between surgeons’ satisfaction and country-specific engagement in digital transformation. Moreover, more than 80% of the participants stated to work at an academic institution, which may distort the actual prevailing availability of access to digital solutions, and thus also not reflect the current reality in thoracic surgical departments. Regional hospitals might have restricted possibilities for digital transformation in their department as compared to the respondents working in academic centres.

Another limitation, reducing the representativeness of the study, was the low response rate to our survey: 6.3% of responses were lower than seen in most previous ESTS surveys and significantly lower than reported in patient and health care professional surveys in surgery [10, 29–31]. This low return can be partially explained by the fact that neither email reminders have been sent nor has an advertisement campaign on the survey been launched. Moreover, this survey was conducted using digital tools and it is likely that ESTS members with low affinity to digitalization did not respond. This might have created a sampling bias, in addition, to ultimately affecting the outcomes of the survey. When compared to one previously published ESTS survey, participation was equally low in the survey by Pompeo *et al.* on non-intubated thoracic surgery, another less explored field in thoracic surgery [32]. We believe that the rather abstract topic of ‘digital transformation’ might have led to a reserved survey participation amongst ESTS members. A selection bias due to the low response rate may be present, hence the validity, drawn from the conclusions of the survey might be limited.

Moreover, the questionnaire underlying the survey was designed by experts in the field of the ESTS Working Group for Digital Transformation. However, a more thorough Delphi consensus process for developing and pre-testing the questions would have made the design of the survey more robust.

Finally, digital transformation is currently still in its infancy, therefore, we need to monitor future developments and outcomes of patient care and surgical training to draw a conclusion on the importance and influence of thoracic surgery.

## CONCLUSION

Our survey shows the regular application of digital solutions in Thoracic Surgery and emphasizes the importance of Digital Transformation in Thoracic Surgery in the presence of about two-thirds of the participating ESTS members being satisfied with their current digital engagement.

The ESTS Digital Transformation Working Group will keep working on their mandate to further promote the digital transformation of the Thoracic Surgery landscape.

## Supplementary Material

ivae119_Supplementary_Data

## Data Availability

The data underlying this article will be shared on reasonable request to the corresponding author.

## ABBREVIATIONS

COVID-19: Corona virus disease 2019
ESTS: European Society of Thoracic Surgeons
GDPR: General Data Protection Rating
