# Supplementary material for: Digital Transformation in Thoracic Surgery: a survey among the European Society of Thoracic Surgeons
Source: Interdiscip Cardiovasc Thorac Surg. 2024 Jun 28;39(1):ivae119. doi: 10.1093/icvts/ivae119 (PMC11222297; doi:10.1093/icvts/ivae119)
Supplement: ivae119_Supplementary_Data [file ivae119_supplementary_data.pdf]

Supplementary 1 – Original survey

2022 Digital Transformation in Thoracic Surgery – an ESTS Survey
A survey by the ESTS-Digital Transformation Working Group
Powered by SurveyMonkey

Opened: March 13, 2022 to
Closed: May 21, 2022

Q1: In what country do you work?

Q2: What is your age?

Q3: What is your gender?

Q4: For how long have you been in practice as a consultant/staff surgeon?

Q5: Please indicate your background training:
a. General surgery
b. Thoracic surgery
c. Cardiothoracic surgery
d. Thoraco-vascular surgery
e. Other, please specify

Q6: In which type of institution do you practice?
a. Academic hospital
b. Supraregional, non-academic hospital
c. Regional, non-academic hospital
d. Other, please specify

Q7: How would you rate the digital transformation in Thoracic Surgery?
a. Not important
b. Somewhat important
c. Important
d. Very important
e. Fundamental

Q8: Are you currently using any digital platform for direct patient care?
a. Yes
b. No

Q10: If you answered YES to Question 8 (Are you currently using any digital platform for direct patient care?), where do you see the best value of your digital platform?

a. None of the below mentioned
b. Pre-assessment
c. Hospital stay
d. Post-operative care
e. All the above

Q11: For your clinical studies, do you extract your data from: (more than one answer accepted)

a. A local database (Excel, Google drive etc.)
b. Hospital database
c. National database
d. ESTS database
e. Other (please specify)

Q12: Are you currently using any digital platform for training and education?

a. Yes
b. No
c. I do not know

Q13: If NO to question 13 (are you currently using any digital platform for training and education?), would you consider introducing one in your department?

a. Yes
b. No

Q14 (part 1): If you answer YES to question 13 (are you currently using any digital platform for training and education?) are you mostly using in:

a. Virtual teaching and/or Symposium
    a. Never
    b. Rarely
    c. Sometimes
    d. Often
    e. Usually
    f. Always
b. Face to face teaching and/or Symposium
    a. Never
    b. Rarely
    c. Sometimes
    d. Often
    e. Usually
    f. Always
c. Hybrid (both of the above) Teaching and/or Symposium
    a. Never
    b. Rarely
    c. Sometimes
    d. Often
    e. Usually

f. Always

Q14 (part 2): If you answer YES to question 13 (are you currently using any digital platform for training and education?) are you mostly using in:

a. Virtual surgical training
    a. Never
    b. Rarely
    c. Sometimes
    d. Often
    e. Usually
    f. Always
b. Hands on surgical training
    a. Never
    b. Rarely
    c. Sometimes
    d. Often
    e. Usually
    f. Always
c. Hybrid (both of the above) Surgical Training
    a. Never
    b. Rarely
    c. Sometimes
    d. Often
    e. Usually
    f. Always

Q15: If you answered YES to question 13 (are you currently using any digital platform for training and education?) are you mostly using in:

a. Virtual teaching and/or Symposium
    a. Never
    b. Rarely
    c. Sometimes
    d. Often
    e. Usually
    f. Always
b. Face to face teaching and/or Symposium
    a. Never
    b. Rarely
    c. Sometimes
    d. Often
    e. Usually
    f. Always
c. Hybrid (both of the above) Teaching and/or Symposium
    a. Never
    b. Rarely
    c. Sometimes
    d. Often
    e. Usually
    f. Always
d. Virtual surgical training

    a. Never
    b. Rarely
    c. Sometimes
    d. Often
    e. Usually
    f. Always
e. Hands on surgical training
    a. Never
    b. Rarely
    c. Sometimes
    d. Often
    e. Usually
    f. Always
f. Hybrid (both of the above) Surgical Training
    a. Never
    b. Rarely
    c. Sometimes
    d. Often
    e. Usually
    f. Always

Q16: Are you currently using any digital platform for networking and communications?

a. Yes
b. No

Q17: If you answered YES at Question 16 (Are you currently using any digital platform for networking and communications?) with what frequency are you using it:

a. Multidisciplinary board discussion with other colleagues
    a. Never
    b. Rarely
    c. Sometimes
    d. Often
    e. Usually
    f. Always
b. Virtual Meeting National or International
    a. Never
    b. Rarely
    c. Sometimes
    d. Often
    e. Usually
    f. Always
c. Social Networking (LinkedIn, Twitter (“X”), Facebook…)
    a. Never
    b. Rarely
    c. Sometimes
    d. Often
    e. Usually
    f. Always

d. Work communication between colleagues
    a. Never
    b. Rarely
    c. Sometimes
    d. Often
    e. Usually
    f. Always
e. Research Meeting
    a. Never
    b. Rarely
    c. Sometimes
    d. Often
    e. Usually
    f. Always

Q18: What is the app for social networking that you mostly use? Multiple answers are allowed.

a. LinkedIn
b. Facebook
c. Twitter (“X”)
d. Instagram
e. Telegram
f. Other (please specify)

Q19: Are you using any institutional app for work communication between colleagues?

a. Yes
b. No (I mostly use… *please specify in the comment field*)
c. Not at all

Q20: Would you consider a specified digital platform for healthcare professional?

a. Yes
b. No
c. I do not know

Q21: How much are you satisfied with your digital transformation engagement currently?

a. Very satisfied
b. Satisfied
c. Somewhat satisfied
d. Neither satisfied nor dissatisfied
e. Somewhat dissatisfied
f. Dissatisfied
g. Very dissatisfied

Q22: Where do you think this ESTS group should be focusing on?

a. Introduction of digital platforms for patient healthcare
b. Introduction of digital platforms for teaching and training

c. Introduction of digital platforms for clinical communication and social media between colleagues
d. Other (please specify)

Q23: Open space for comments.
